# Virtual Screening of Specific Insulin-Like Growth Factor 1 Receptor (IGF1R) Inhibitors from the National Cancer Institute (NCI) Molecular Database

**DOI:** 10.3390/ijms131217185

**Published:** 2012-12-14

**Authors:** Cong Fan, Yan-Xin Huang, Yong-Li Bao, Lu-Guo Sun, Yin Wu, Chun-Lei Yu, Yu Zhang, Zhen-Bo Song, Li-Hua Zheng, Ying Sun, Guan-Nan Wang, Yu-Xin Li

**Affiliations:** 1 National Engineering Laboratory for Druggable Gene and Protein Screening, Northeast Normal University, Changchun 130024, China; E-Mails: fanc232@nenu.edu.cn (C.F.); sunlg388@nenu.edu.cn (L.-G.S.); wuy705@nenu.edu.cn (Y.W.); yucl885@nenu.edu.cn (C.-L.Y.); zhangy259@nenu.edu.cn (Y.Z.); songzb484@nenu.edu.cn (Z.-B.S.); zhenglh015@nenu.edu.cn (L.-H.Z.); suny040@nenu.edu.cn (Y.S.); cabthy@163.com (G.-N.W.); 2 Research Center of Agriculture and Medicine Gene Engineering of Ministry of Education, Northeast Normal University, Changchun 130024, China

**Keywords:** IGF1R, IR, virtual screening, binding mode prediction, selective inhibition

## Abstract

Insulin-like growth factor 1 receptor (IGF1R) is an attractive drug target for cancer therapy and research on IGF1R inhibitors has had success in clinical trials. A particular challenge in the development of specific IGF1R inhibitors is interference from insulin receptor (IR), which has a nearly identical sequence. A few potent inhibitors that are selective for IGF1R have been discovered experimentally with the aid of computational methods. However, studies on the rapid identification of IGF1R-selective inhibitors using virtual screening and confidence-level inspections of ligands that show different interactions with IGF1R and IR in docking analysis are rare. In this study, we established virtual screening and binding-mode prediction workflows based on benchmark results of IGF1R and several kinase receptors with IGF1R-like structures. We used comprehensive analysis of the known complexes of IGF1R and IR with their binding ligands to screen specific IGF1R inhibitors. Using these workflows, 17 of 139,735 compounds in the NCI (National Cancer Institute) database were identified as potential specific inhibitors of IGF1R. Calculations of the potential of mean force (PMF) with GROMACS were further conducted for three of the identified compounds to assess their binding affinity differences towards IGF1R and IR.

## 1. Introduction

Insulin-like growth factor 1 receptor (IGF1R) is a transmembrane tyrosine kinase that is widely found in many cell types and is essential for normal fetal and postnatal growth and development [1]. IGF1R is composed of two extracellular α-subunits bound to a transmembrane-spanning β-subunit through a disulfide bond. The cytoplasmic tyrosine kinase domain is located in the β-subunit. The kinase catalytic ability is activated through phosphorylation at specific Tyr residues [1] after structural rearrangements caused by the binding of IGF1 or IGF2 to the ectodomain of IGF1R. Phosphorylation leads to conformational changes that provide a binding site for diverse proteins that initiate signaling cascades. These signaling cascades are mainly involved in proliferation and protection from programmed death. Therefore, IGF1R is a regulator that is vital to growth, differentiation and apoptosis. Attention to IGF1R has been increasing since the end of the 1980s because of growing evidence that IGF1R-mediated signaling is crucial for the development and progression of multiple types of cancer. The antitumor activity of IGF1-IGF1R signaling inhibitors has been examined since 2000. Small-molecule inhibitors and antagonistic monoclonal antibodies against IGF1R have shown results in clinical trials. By 2010, clinical research on several drug candidates reached Phase III [2]. Current potential IGF1R inhibitors can be grouped into three main types. The first group is ATP-competitive inhibitors, which block ATP binding to IGF1R, preventing kinase activation. This group includes tyrphostins [3], pyrrolopyrimidines and pyrazolopyrimidines [4], benzimidazole-pyridones [5], imidazopyrazines [6], and others. Some compounds in this first group show selectivity for IGF1R against other similar kinase receptors including insulin receptor (IR) [2]. The second group is ATP-noncompetitive inhibitors, including picropodophtllin [7], AG538 [8] and SBL02 [9]. The main inhibition mechanism of these ligands is competing with IGF1R substrates rather than competing with ATP binding. The third group contains inhibitors whose mechanism is unknown or that work indirectly. This group includes simvastatin [10] and inhibitors of heat shock protein 90 (HSP 90) [11], whose inhibition decreases the number of IGF1R molecules in the plasma membrane.

Although many small-molecule inhibitors of IGF1R have been discovered, key problems remain, including the identification of inhibitors specific to IGF1R rather than IR. The kinase protein IR shares high sequence similarity to IGF1R but has a different role in development. Thus, inhibition of IR by IGF1R inhibitors might lead to risks. Compounds that show selective inhibition of IGF1R over IR have been discovered through experimental methods such as identification of a novel class of pyrrole-5-carboxaldehyde compounds by Bell *et al.* in 2005 [12]. Computational methods have been introduced to solve the specificity problem. In 2010, a new class of IGF1R-selective inhibitors was discovered by Krug *et al.* through experimental methods that included computer-aided docking analysis [13]. Also in 2010, Liu *et al.* identified two thiazolidine-2,4-dione analogs as potent and selective IGF1R inhibitors with the aid of hierarchical virtual screening and SAR (structure-activity relationship) analysis [14]. Jamakhani *et al.* generated three-dimensional structures of IGF1R using homology modeling and identified IGF1R inhibitors via molecular docking, drug-like filtering and virtual screening [15]. However, rapid identification of new lead compounds as potential selective IGF1R inhibitors through receptor structure-based virtual screening and inspection of differences in ligand interactions with IGF1R and IR through docking analysis are rare. Here, we designed and built computational workflows to solve these problems. In this study, a virtual screening workflow was established using benchmark results from docking software analysis of seven kinase proteins with structures highly similar to IGF1R. Experimentally proven inhibitors and decoy inhibitors were carefully extracted from the DUD database [16]. Effects of this workflow were further tested on IGF1R with another ligand set, and the results showed that known inhibitors of IGF1R were ranked by statistical significance ahead of randomly selected ligands. With the aid of this workflow, 90 of 139,735 compounds in the NCI database were selected as potential inhibitors of IGF1R [17]. To further investigate the inhibition selectivity of these compounds, we created a binding-mode prediction workflow that correctly predicted the binding modes of the ligands for IGF1R and IR, based on comprehensive analysis of known complexes of IGF1R and IR with their binding ligands. With this workflow, we generated and inspected the binding modes of 90 previously selected compounds against IGF1R and IR. As a result, 17 compounds were identified as inhibitors specific to IGF1R and not IR. Among these, three showed the best inhibition potency, and the calculations of the potential of mean force (PMF) with GROMACS were further conducted to assess their binding affinity differences towards IGF1R and IR. Checking the compounds selected from NCI with our workflows with results published by the Developmental Therapeutics Program (DTP) [17], showed that most of the selected compounds had growth inhibition effects on many human tumor cell lines. The inhibitory activity of these identified ligands for IGF1R *in vitro* or *in vivo* requires further experimental verification.

## 2. Results

### 2.1. Virtual Screening Workflow

**Figure 1:**
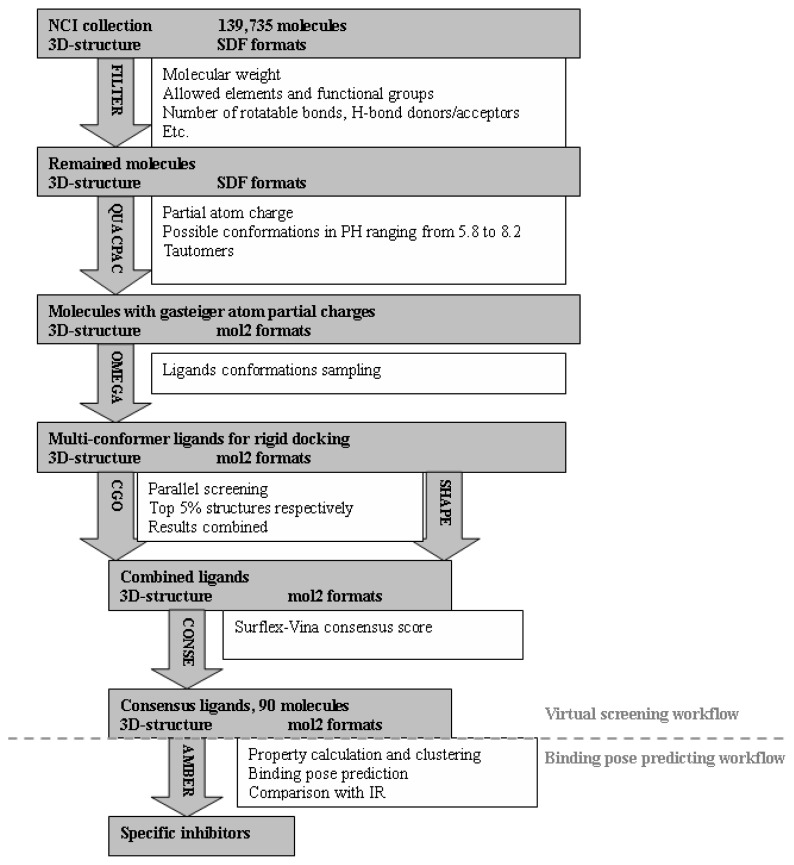
The flow chart of the virtual screening workflow.

Score functions in popular, free, academic software were chosen as candidate components for a virtual screening workflow to identify IGF1R inhibitors. The functions were forcefield-based grid scores in DOCK [18], empirical scores in Surflex [19] and FRED [20], and semi-empirical scores in Autodock [21] and Autodock Vina [22]. A virtual screening workflow was built after a series of tests and statistical analyses of docking results for seven kinase receptors with structures similar to IGF1R and their corresponding ligand sets from the DUD database [16] (Figure 1). The workflow was designed to have two rounds of screening. The first round decreased the size of the compound pool, and the second selected IGF1R inhibitors. Details about software setup in the workflow can be found in the experimental section.

**Figure 2:**
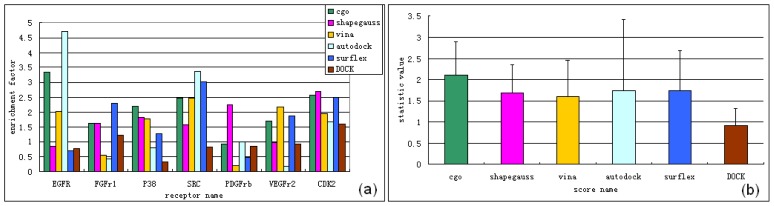
Known-binding-ligands-enrichment ability of chosen software against seven kinase receptors. Statistic analysis was performed in top 20% of the rank. (**a**) enrichment factors (EFs) of ligands docking to seven kinase receptors by chosen softwares; (**b**) Average value and SD of software’s docking EFs (taking from Figure 2a) among seven kinase receptors.

A combination of both cgo and shapegauss score functions in FRED was used in the first round of virtual screening, because the two score functions were the fastest and had relatively consistent performance for the seven chosen receptors. As listed in Table 1, the average time for each molecule was calculated and the total time for 100,000 (close to the number of compounds in the NCI database) was predicted for each software tool. Table 1 shows that FRED performed much faster than the other tools. Performance comparisons for each score function are in Figure 2. We concluded that the FRED cgo score performed more stably and better than other docking packages for the seven kinase protein targets. This led to the highest average enrichment factor (EF) of 2.12 (calculation of EF is in section 3.1) and a low standard deviation (SD) of 0.78 (Figure 2a,b). Although the shapegauss score did not perform as well as the cgo score, yielding a lower average EF score of 1.68, it appeared out a complementary ability of enriching known active ligands with the cgo score. This was reflected most clearly by docking with EGFr, SRC, PDGFrb and VEGFr2. Therefore, we were confident that the combination of the cgo and shapegauss score functions would be a rapid and effective tool to reduce the size of a large compound pool. Through enrichment distribution analysis, we found that both of the two score functions enriched known active ligands most densely within the top 1% of the rank. To insure that enough hits could be contained in the reduced compound pools, the top ranked 5% from both scores in practical applications were to combined, causing the size of molecular pool reduced to not more than one tenth.

The reduced compound pool was used for the second round of virtual screening, which used the Surflex and Vina consensus scores. Both the Surflex and Vina score functions performed well in most cases and the highest number of consensus results was obtained using these two scores. Figure 2a shows that none of the chosen score functions effectively enriched known active ligands for the receptors. This was in accordance with current benchmarking studies showing that no score always performed well for every receptor [23]. Structural flexibility makes kinase receptors a challenging protein target for current docking software according to the DUD [16]. To solve difficulties in enriching potential inhibitors, we adopted the principle of choosing the consensus results of the top-ranked results from two reliable score functions as our final selection [23]. Figure 2b shows that AutoDock gave higher average EF values than Surflex, Vina or DOCK, but its SD was as high as 1.69, indicating an unstable performance. Figure 2a shows that Autodock provided good enrichment capabilities against the kinase receptors EGFr and SRC. However, the results were relatively poor for other receptors (FGFr, P38, VEGFr2 and PDGFrb). The grid score from DOCK had the smallest SD, but did not give the best performance for any of the chosen receptors, resulting in the lowest average EF of 0.92. Comparing the four docking packages, Surflex gave the best enrichment capabilities for the receptors FGFr1 and CDK2, with modest enrichment of the receptors P38, SRC and VEGFr2 and poor enrichment of EGFr and PDGFrb. This led to an average EF of 1.73 and an acceptable average SD of 0.96. For Vina, low EFs were seen for the receptors FGFr1 and PDGFrb, but high, stable enrichment was seen for the five other receptors. This resulted in an average EF of 1.59 and an acceptable average SD of 0.86 (Figure 2a,b). Although Vina did not perform as well as Surflex, increasing the exhaustiveness parameter value improved its performance for a given receptor (data not shown). Both Vina and Surflex were fast (Table 1). Based on these results, we chose the Surflex and Vina consensus scores for the second round of virtual screening. Using each software package, distributions of enriched active ligands were calculated. Detailed results are shown in Figure S1 of Supplementary File.

**Figure 3:**
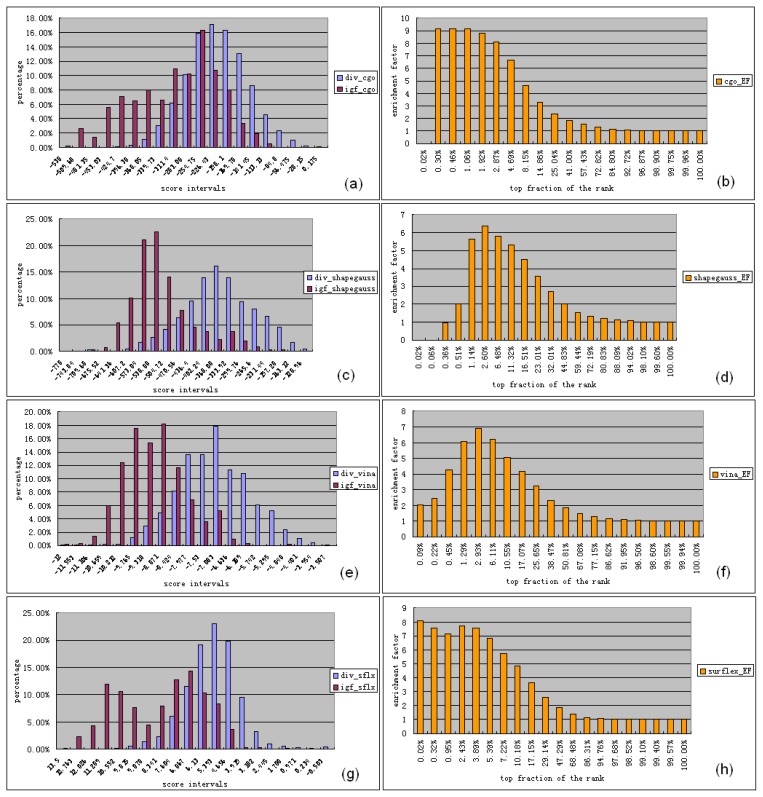
Score distributions of known inhibitors and random ligands generated by (**a**–**b**) cgo, (**c**–**d**) shapegauss, (**e**–**f**) Vina and (**g**–**h**) Surflex. Results of known inhibitor collections were recorded as “igf” and that of random ligands or diversity set III were recorded as “div”.

Because IGF1R was not in the receptor target set of the DUD database, we constructed a test dataset for IGF1R to evaluate workflow performance. The test dataset had two parts: one for known IGF1R inhibitors and the other for random ligands. Score distributions from FRED (cgo and shapegauss), and Vina and Surflex were checked by virtual screening. Figure 3 shows percentages of known inhibitors and random ligands for each score interval of the score functions. The score distributions generated by cgo, shapegauss and Vina all fit to a normal distribution. Therefore, a student’s *t*-test was carried out for a confidence interval of 99%. The results confirmed that known inhibitors scored significantly better than random ligands when Vina, cgo and shapegauss were tested. To further inspect the enrichment capabilities of each score function, we studied the relationship between EFs and top fractions of the ranked compounds. Figure 3 shows that for the cgo score, EFs were maintained at higher than 6.0 in the range of 0.3%–4.69% of the top-ranked molecules. For the shapegauss score, EFs were higher than 5.0 in the range of 1.14%–11.32% of the top-ranked compounds. Therefore, both cgo and shapegauss scores worked well for the first round of virtual screening for IGF1R. Vina exhibited similar score distributions as shapegauss, with EFs at levels higher than 5.0 in the range of 1.29%–10.55% of the top ranked molecules. For Surflex, score distributions of known inhibitors exhibited two peaks (Figure 3e–h), unlike the random ligands, which exhibited normal distributions. The EFs for known inhibitors in the first peak reached levels higher than 6.0. In summary, although the EFs in the top 10.18% of the ranked molecules were higher than 4.85 using Surflex (Figure 3h) and slightly lower than 5.0, enough potential IGF1R inhibitors were enriched into the top 10% by Vina and Surflex. Diversity set III [17], which was treated as the collection of random ligands, was a representation of pharmacophores of the entire NCI database we planned to screen. Therefore, the score distributions of the dataset of random ligands for each score function reflected the results from the NCI database to some degree. Overlaps in score distribution were seen between the known inhibitor set and the diversity set for each score function. This suggested a high chance that a number of potential IGF1R inhibitors were in the NCI database (Figure 3a,c,e,g). These results indicated that the assembled virtual screening workflow performed well in identifying potential IGF1R inhibitors.

### 2.2. Binding Mode Predicting Workflow

**Figure 4:**
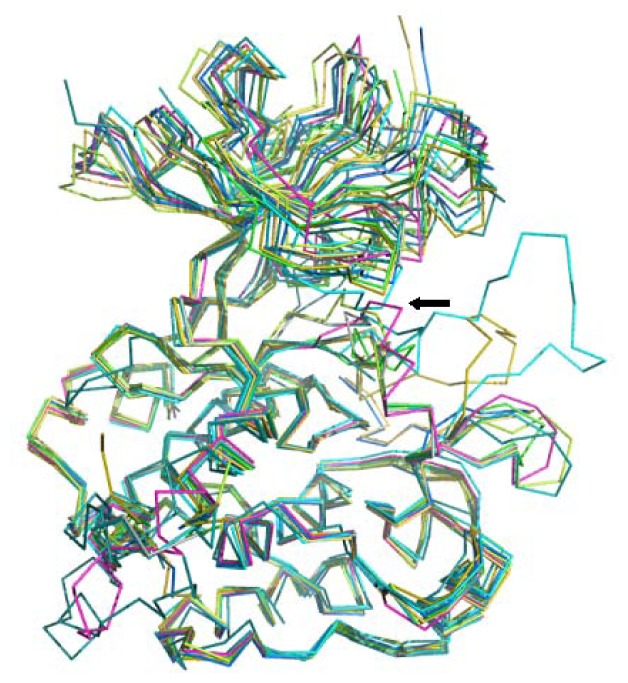
Superimposition of nine PDB crystal structures of Insulin-like growth factor 1 receptor (IGF1R). Picture was made by Pymol V0.99 [25]. The active site focused here is locating in the cave formed by lobes respectively near the *N*- and *C*-terminals. It can be seen from the picture that the regions near the focused active site are of high flexibility (indicated by a black arrow).

**Figure 5:**
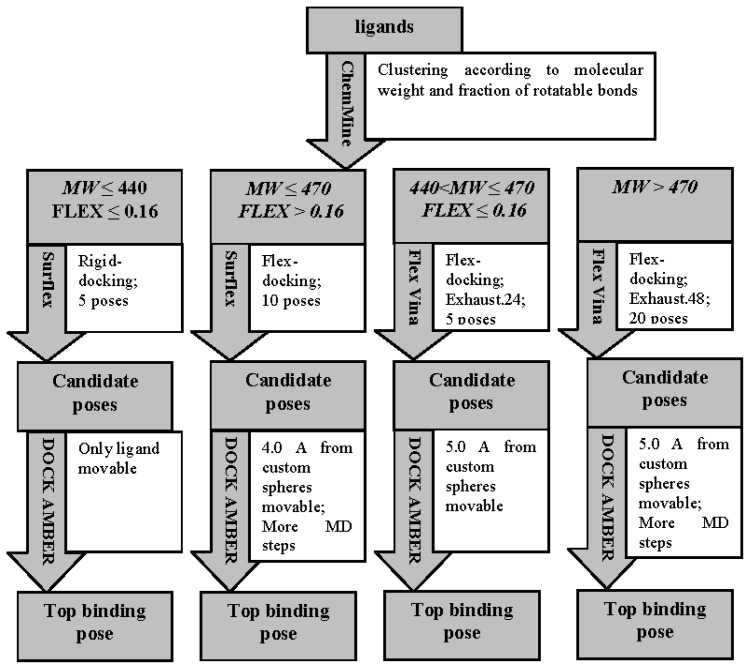
The flow chart of the binding mode predicting workflow.

We collected nine nonredundant crystal structures of complexes of human IGF1R with binding ligands from the PDB database [24]. Superposition of the structures showed high flexibility in the ATP-binding site of IGF1R (Figure 4). Backbone movements upon binding of some ligands makes it difficult to predict ligand-binding positions compared to rigid active sites because docking software packages treat receptor backbones as rigid. Calculating and analyzing the physiochemical characteristics of crystal structures of ligands in the nine complexes showed great variation in the molecular sizes and rotatable bond numbers of the binding ligands. This made predicting the binding position challenging. For this reason, we found few software programs that could predict ligand binding positions correctly. To gain more accuracy, we assembled a multisoftware docking workflow (Figure 5).

**Figure 6:**
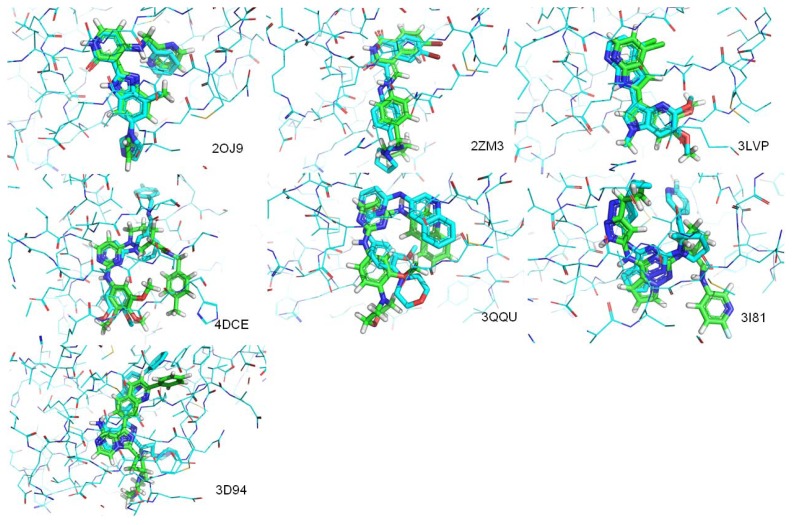
Comparisons of crystal and predicted poses of ligands from seven IGF1R complexes. Pictures were made by Pymol V0.99 [25]. Atoms were colored according to their type. Cyan lines represents protein residues of IGF1R, cyan sticks represents crystal poses of ligands, and green sticks represents predicted pose of ligands.

We clustered the nine ligands into four groups according to molecular weights (*MW*) and rotatable bond numbers (FLEX) of the binding ligands (Table 2). The first group contained ligands with *MW* ≤ 440 and FLEX ≤ 0.16 (calculation of *MW* and FLEX is in Section 3.3). This group is represented by the crystal structure of ligands 2OJ9, 2ZM3 and 3LVP. This type of ligand favors an extended position that is not deep in the active site. The small size of these ligands means the receptor does not need large alterations in conformation (Figure 6). The second type of ligand showed high flexibility and a small size with *MW* ≤ 470 and FLEX > 0.16. This type contained the crystal structure of ligands 4DCE, 3QQU and 3I81. This ligand type tended to bind shallowly in the active site in a folded position that did not occupy much of the active site (Figure 6). The third ligand type had a small *MW* (>440 and ≤470) and low FLEX (≤0.16), such as crystals structure of 3D94 and 3O23. For this type of ligand, a not-small size and rigid structure favored a large space in the active site and deep positioning with an extended structure (Figure 6). The fourth group contains ligands with *MW* > 470, represented by crystal ligands of 3F5P. This type of ligand possessed a high molecular weight and high flexibility, such as 3F5P with a FLEX value of 0.293. This type of flexible ligands appeared to be large enough that protein backbone movements might be required to accommodate the ligand, and molecular dynamics (MD) techniques might be needed to solve this type of flexible docking problem. Figure 5 shows that for the first group, a rigid docking method was used to predict the binding mode. For the second group, during position-generating, the receptor was kept rigid. However, more positions were used for selection, flexibility was introduced into the receptor, and during calculations of DOCK AMBER, MD steps were increased. For ligands in the third group, high side-chain flexibility around the active-site residues was needed, even though the backbone rather than the side chains needed to be movable to change the active site conformation. Vina that allowed side chains around the active-site residues to move was used to generate possible positions for the third group of ligands. DOCK AMBER with more receptor flexibility was introduced to increase the chance that correct conformations would appear in the top ranking. Finally, we assumed that the last group of ligands would require more space in the active site, so their binding modes were predicted by a flexible mode of Vina with DOCK AMBER. The exhaustiveness parameter value was doubled for Vina to increase the likelihood to top-ranking reasonable poses of ligands with high flexibility that required additional optimization steps.

Crystal structures of ligands were extracted and re-docked to IGF1R using our workflow. In-place RMSD (root-mean-square deviation) was calculated to evaluate atom distances between the crystal structure conformation and the predicted conformation for each ligand. Rigid docking yielded sufficiently informative results for the first ligand group, with RMSD values between re-docked ligands and crystals that were all less that 1.3 ångstroms (Table 2). In the second group of ligands, for 3QQU, the RMSD value was less than 2.0 ångstroms, indicating a correctly predicted binding position for the entire ligand. RMSD values for 4DCE and 3I81 were 4.071 ångstroms and 3.836 ångstroms, considered failed dockings. However, we accepted these ligands because we found that the dockings for most parts of the ligands were positioned correctly. Visual inspection showed that the orientations of the key pharmacophoric interactions were also correctly placed. Furthermore, for these two ligands, high RMSD values resulted from side fragments extending in an incorrect direction rather than a mismatch of the entire structure (Figure 6). These situations have been encountered in other studies that accepted these types of dockings as roughly correct [26]. For 3D94, in the third group, whose FLEX was less than 0.1, the correct binding position had an RMSD value of 1.512 ångstroms (Table 2). For 3O23, also in the third group, correct structures were generated by Vina, but were not identified by DOCK AMBER. A correct binding model could not be generated for the only ligand in the fourth group.

**Figure 7:**
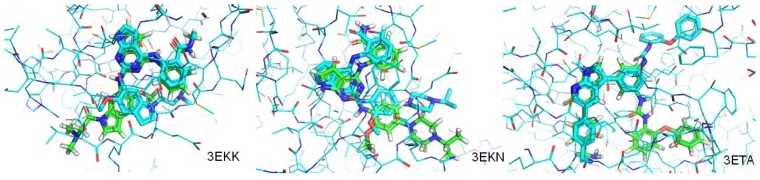
Comparisons of crystal and predicted poses of ligands from three 1R complexes. Pictures were made by Pymol V0.99 [25]. Atoms were colored according to their type. Cyan lines represents protein residues of 1R, cyan sticks represents crystal poses of ligands, and green sticks represents predicted pose of ligands.

We collected four additional crystal structures of ligands that complexed with IR from the PDB database and re-docked them into the ATP-binding site of IR to evaluate the trained binding-predicton workflow (Figure 7). In-place RMSD between the crystal structure conformation and the re-docked conformation for each ligand was calculated as in Table 3. Three of the four ligands were predicted correctly with in-place RMSD values less than 4.10. These results indicated the workflow worked well for predicting binding modes to IR.

In summary, our binding mode-predicting workflow effectively predicted ligand positioning in the ATP-binding sites of IGF1R and IR. Using this workflow, we examined the characteristics of the interactions between each ligand and IGF1R/IR, such as interacting atoms and binding affinities. Thus, this workflow was used to analyze potential specific inhibitors of IGF1R by comparing ligand-IGF1R/IR interactions in detail.

### 2.3. Screening Potential Specific Inhibitors of IGF1R from the NCI Database

The NCI/DTP Open Chemical Repository [17] contains synthetic compounds and pure natural products. The repository, maintained by DTP, supplies samples for nonclinical investigations. For the 139,735 compounds in the NCI database, 11,504 compounds were detected in the first round of virtual screening. After the second round of virtual screening, 90 compounds were identified for the binding mode-predicting workflow for specific inhibitors of IGF1R. Scores of the selected ligands were calculated for each step. Ligand scores selected by cgo were −476 to −310; by shapegauss were −785 to −503; by Surflex were 12 to 8.24; and by Vina were −12 to −9.6. These results were similar to the scores of known inhibitors of IGF1R (Figure 3).

**Figure 8:**
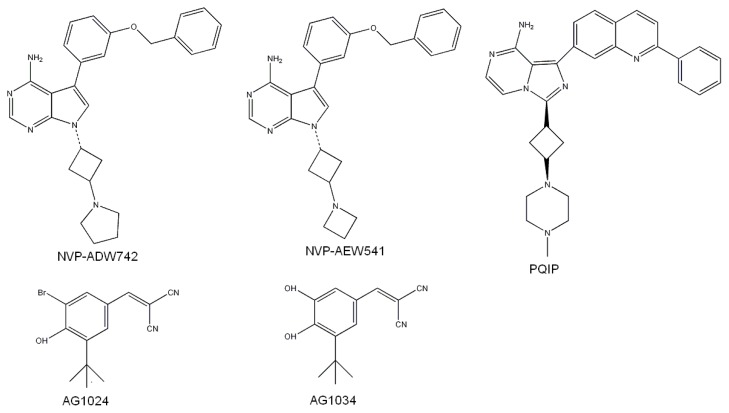
2-D structures of reported five IGF1R specific inhibitors.

**Figure 9:**
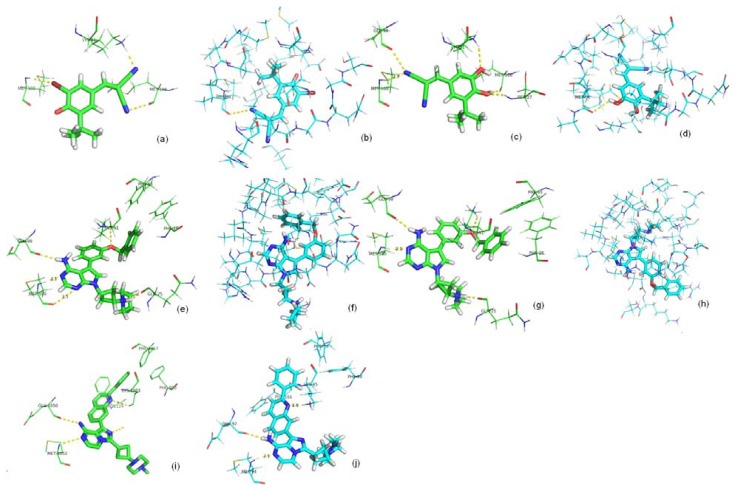
Comparisons of binding poses of reported selective inhibitors against IGF1R and IR. As shown in (**a**–**j**), atoms were colored according to their type. Protein residues were represented by lines and ligands were represented by sticks. Green lines and sticks exhibit ligands’ binding poses in IGF1R active site, while cyan ones exhibit ligands’ binding poses in IR active site. Yellow dash lines indicate H-bonds automatically exhibited by Pymol while the one with a number on it indicate H-bond that was not identified by Pymol V0.99 [25].

Although IGF1R and IR share almost 100% sequence identity in the ATP-binding site, the receptors have differences, probably in local conformation [27]. To explore the selective inhibition mechanisms, we simulated the binding modes of five published selective inhibitors of IGF1R, using the binding mode-prediction workflow (Figure 8). The first two inhibitors, AG1024 and AG1034 which belong to Tyrphostins, had been reported to show significant inhibition selectivity on IGF1R against IR [28]. The docking results showed two possible binding modes that seemed equally reasonable (Figure 9a,c). For AG1024, a single H-bond was formed between a Br atom and the NH group of the Met100 residue, while another H-bond was formed between the N atom of the CN group and the Lys51’ NH3 group (Figure 9a). A different possibility was seen for AG1034 in which the N atom of the CN group formed H-bonds with residues Met100 and Glu98, which are located in the connecting section of the *N*- and *C*-terminal lobes. In addition, the O atom of the OH group formed an H-bond with Lys51 in the *N*-terminal lobe (Figure 9c). Although these inhibitors had two different binding possibilities, we still detected common characteristics of their binding modes. We concluded that H-bonds between polar atoms of the ligands and Met100 and Lys51 were crucial for stabilizing the complex structure. These types of key ligand-receptor interactions have been seen previously. H-bonds between Glu98/Met100 of IGF1R and ligands have been discovered not only in the ATP-IGF1R complex but also for about 10 other inhibitors [29–35]. H-bonds between Lys51 of IGF1R and ligands have not been extensively investigated but could still be important [12]. In docking with IR, we found that ligands could not be reasonably positioned for formation of these H-bonds (Figure 9b,d). Two other inhibitors, NVP-ADW742 and NVP-AEW541, showed >16-fold and 27-fold greater inhibition of IGF1R than IR [2], with comparable agreement in binding modes by our docking models (Figure 9e,g). N and NH2 groups on the purine base of each ligand formed three H-bonds with Glu98 and Met100, and the O-atom formed an H-bond with Lys51. Moreover, we observed hydrophobic interaction between the phenyl group of NCP-AEW541 and the side chains of Phe65/Phe28 within the C-lobe. The same kind of interactions was seen for NVP-ADW742. Hydrophobic interactions have been reported in the crystal structure of complex of IGF1R and the inhibitor PQIP [33]. Therefore, we concluded that hydrophobic interactions between Phe65/Phe28 side chains and nonpolar groups of ligands are also important for stabilizing the receptor-ligand binding state. However, when docked to IR, these NVP-ADW742 and NVP-AEW541 showed incorrect positioning for the formation of these interactions (Figure 9f,h). Another inhibitor, PQIP, is reported to have 14-fold more selective inhibition of IGF1R than IR, and the crystal structure of the complex of PQIP and IGF1R is published (Figure 9i) [33]. However, we obtained a mode of PQIP binding to IR that was very similar, with the only difference in the deep part of the IR active site. The three Phe residues did not form a clear hydrophobic interaction with the nonpolar groups of PQIP, while IGF1R did (Figure 9j) [33]. Therefore, the cause of the selective inhibition of this type of ligand was still unknown. Based on our results, we concluded that H-bonds between ligands and the NH3 group of Lys51 and residues in the lobe-connecting sections such as Met100 and Glu98 were important to the high affinity of the IGF1R inhibitors. Hydrophobic environments around Phe65 and Phe28 were also important for containing the lipophilic groups of ligands and stabilizing the complex structure. However, the conformation of the entire kinase domain of IR might make proper interaction with some of the compounds difficult. These were the compounds that exhibited highly selective inhibition against IGF1R rather than IR.

**Figure 10:**
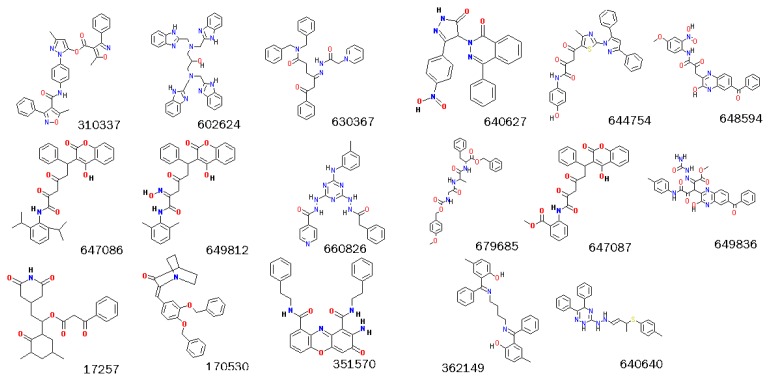
2-D structures of 17 identified potential IGF1R specific inhibitors. N atoms were colored blue and O atoms were colored red. C atoms were kept in black.

Based on our observations, we identified compounds as potential selective inhibitors of IGF1R if they formed at least one H-bond with Lys51 or Glu98/Met100 of IGF1R and had a ring structure near residues 98–100, while showing none of the these interactions with IR. Compounds that formed H-bonds with Lys51 and Glu98/Met100 in parallel should be considered strong potential inhibitors of IGF1R. Compounds that exhibit hydrophobic interactions with Phe65/Phe28 as well as H-bonds with Lys51 and Glu98/Met100 should also be considered as powerful IGF1R inhibitors. According to these rules, 17 of 90 compounds were selected as potential specific inhibitors of IGF1R (Figure 10). Among these, three compounds (351570, 660826 and 649812) were considered as very powerful inhibitors of IGF1R.

Tens of thousands of compounds have been analyzed by the DTP Human Tumor Cell Line Screen for growth inhibition of human cancer cell lines [17]. Using NCI data, we confirmed that 29 of the 90 potential inhibitors of IGF1R and 12 of the 17 specific inhibitors of IGF1R showed growth inhibition effects on many human tumor cell lines. The predicted binding modes against IGF1R and IR for the 17 compounds are in Figure S2 in Supplementary File.

### 2.4. Binding Affinity Comparisons for Ligands of IGF1R and IR with PMF Calculations

In MD simulations of a ligand-receptor system, the PMF curve approach can be constructed as a ligand is pulled from the binding site to bulk water. This estimates the free energy change from the bound to the unbound state [36]. We selected three potent compounds (351570, 660826 and 649812) for MD simulations to explore binding affinity differences to IGF1R and IR. Center of mass pulling was employed to calculate receptor-ligand binding free energies ΔGbind. During MD simulations, pulling directions were carefully controlled to make sure that disassociation paths were similar enough to each other [37].

**Figure 11:**
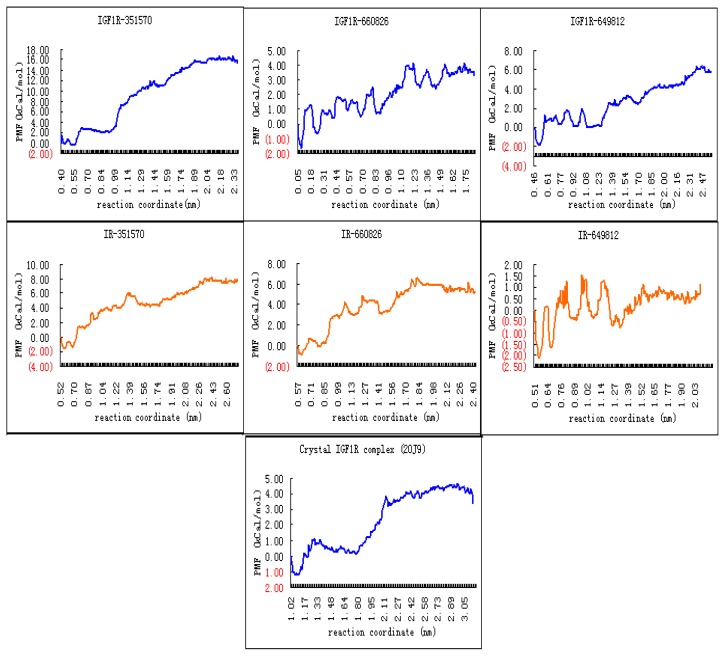
The potential of mean force (PMF) curves of the three compounds complexing with IGF1R and IR and the known ligand of IGF1R (2OJ9).

Figure 11 shows that for compound 351570, the PMF curve of the ligand-IGF1R complex increased as the ligand disassociated shortly after the equilibrating procedure. The curve flattened at 16.5 kCal mol^−1^ when disassociation was completed. For the ligand-IR complex, the PMF curve rose less steeply than for IGF1R, indicating a less stable binding state between IR and compound 351570 than between 351570 and IGF1R. Table 4 shows that compound 351570 showed much higher binding to IGF1R, with a ΔGbind of −13.5 kCal mol^−1^, than to IR with a ΔGbind of −7.2 kCal mol^−1^. Similarly, compound 649812 showed much higher binding to IGF1R, with a ΔGbind of −5.92 kCal mol^−1^, than to IR with a ΔGbind of −0.60 kCal mol^−1^. However, compound 660826 exhibited much higher binding to IR, with a ΔGbind of −5.39 kCal mol^−1^, than to IGF1R with a ΔGbind of −3.09 kCal mol^−1^. Further, the MD simulation was performed on the crystal IGF1R complex (PDB ID: 2OJ9). The binding free energy (ΔGbind) of known ligand of IGF1R was −4.38 kCal mol^−1^. Based on these results, we estimated that compound 649812 may be an ideal, potential specific IGF1R inhibitor, for the ΔGbind of IGF1R-649812 complex is lower than that of crystal IGF1R complex, while the ΔGbind of IR-649812 complex is obviously higher than that of the crystal IGF1R complex. Although compounds 351570 showed much higher binding to IGF1R than IR, the binding free energy of the IR-351570 complex was a little lower than that of the crystal IGF1R complex. Compound 351570 may also stably bind into the active site of IR. In addition, according to DTP results, compounds 660826 and 649812 were tested for ability to inhibit the human cancer cell growth, including non-small cell lung, colon, breast, ovarian, melanoma, leukemia, renal, prostate and central nervous system cells. Inhibition activity of compound 351570 towards various cancer cells has not yet been reported. The *in vitro* or *in vivo* inhibitory activity of these compounds to IGF1R requires experimental verification.

## 3. Experimental Section

### 3.1. Experimental Data Set for Virtual Screening Benchmarking

All protein targets and corresponding ligand sets for virtual screening benchmarking were from the DUD database [16]. DUD offers 40 protein targets with 9 belonging to the group of kinase receptors. We visually examined three-dimensional structures and chose seven proteins whose structures resembled IGF1R. The receptors and PDB IDs were CDK2 (1ckp), EGFr (1m17), FGFr1 (1agw), P38_MAP (1kv2), PDGFrb (model), SRC (2src), and VEGFr2 (1vr2). Ligand and decoy sets established by DUD for each target were adopted in our benchmarking. All ligands and decoys from DUD are prepared through the ZINC database [16,38], so no further preparations were needed for our work. All protein targets from DUD are in PDB format, and water molecules and ions were deleted. Informal residues far from the binding site were ignored, and proton states of certain residues were inspected and adjusted according to Park [39]. AMBER charge were assigned and random H atoms added, followed by a 1000-step dynamic simulation to move only H atoms to more reasonable positions while other parts of the receptors were frozen. This dynamic procedure was performed by GROMACS software [40]. Neither solvent molecules nor ions were added and AMBER03 force field was used.

Known inhibitors of IGF1R were collected from the Binding Database [41,42], and the entire diversity set III was chosen as the random ligand set. Ligands in two-dimensional format in the random ligand set were delivered to QUACPAC of OpenEye [43] to calculate possible proton states for pH values from 5.8 to 8.2. Tautomers of each ligand were also predicted, and gasteiger partial atom charges were added for each generated structure. Finally, structures were transformed from two- to three-dimensional using OMEGA of OpenEye and saved in mol2 format. For known inhibitors, tautomers were not generated, and proton states were conserved, while atom partial charges were displaced by gasteiger. Preparations of receptor files were as for DUD protein targets.

For each receptor, the EF was calculated as below:

(1) EF=(a/n)/(A/N)

Where *a* is the number of known binding ligands in the top-ranked ligands; *n* is the number of the whole ligands (including known binding ligands and decoys) in the top-ranked ligands; *A* is the number of known binding ligands in the whole tested library; and *N* is the number of the whole ligands in the tested library.

### 3.2. Software for Virtual Screening Benchmarking

#### 3.2.1. Surflex-Dock v2.5.1

For each of the seven receptors, the protocol was generated by the location of the crystal structure of the ligand. Docking parameters were: additional starting conformations per molecule and search density were increased to 4; search grid was expanded by 5 ångstroms; conformations per fragment were set to 20 or lower; and the number of rotatable bonds per molecule was 100 or fewer. In addition, the activated spin alignment method was chosen to orient the ligand, with a search density of 4 and number of spins set to 12. For IGF-1R, the starting conformations per molecule and the search density were increased to 6. Scores of known inhibitors and random ligands were abstracted and analyzed by student’s *t*-test.

#### 3.2.2. Autodock v4.2.3

Original structures and partial charges were conserved when generating pdgqt-format files for receptors and ligands. However, for receptors, nonpolar H-atoms were deleted and their partial charges transferred to the attaching atoms. Software default settings were used. The grid box of the active site was centered on the crystal structure of the ligand in the original pdb file, sizing 65 points in all three directions with the grid space set to 0.375 ångstroms. Docking procedures were carried out in parallel, as described by Park [39].

#### 3.2.3. Autodock Vina v1.1.2

The grid box was centered as for Autodock v4.2.3, sizing 26 ångstroms in all three directions, which was almost the same as Autodock v4.2.3. The exhaustiveness parameter was increased to 11 from default 8, to emphasize accuracy over time spent. Other settings were the default. For IGF-1R, scores of known inhibitors and random ligands were abstracted and analyzed by student’s *t*-test.

#### 3.2.4. FRED v2.2.5

For each receptor target, ligands were delivered to OMEGA [44] to generate conformations, adopting default parameters. The file with conformations was sent to FRED for docking. The receptor active site was defined based on co-crystallized ligands instead of grid box, and default docking parameters were used.

#### 3.2.5. DOCK v6.5

Receptor surface files were generated using DMS [45] with radius set to 1.4 ångstroms. SPHGEN [46] was employed to generate spheres to aid in placing ligand atoms, with sphere radii restricted to 0 to 4.0. Spheres within 10.0 ångstroms for any atom of the co-crystallized ligand were used to define the active site, while other spheres were deleted after a visual inspection using CHIMERA graphic software [47]. The grid box was generated using SHOWBOX [48], with 6.5 or 7 ångstroms from the selected spheres enclosed in all six directions. The grid box was set as large as possible to completely cover the open active site formed by two domains, for each of the chosen kinase proteins. “Soft docking” [23] was adopted based on the high flexibility of the active sites, with the repulsive radius set to 9 and bump_overlap set to 0.7 when grids were calculated. Grid scores were employed as the primary score. For additional accuracy, max_orientations, pruning_max_orients and pruning_clustering_cutoff were enlarged to 1000, 150 and 150, respectively. The number of anchors was restricted to 3, while the minimum number of atoms in an anchor was set to 5. Electronic interaction exponents were set as high as 1.8 to establish the polar interaction between the ligands and the kinase receptors.

### 3.3. Ligand-Binding Mode Prediction

#### 3.3.1. Ligand Preparation and Clustering Analysis

Ligands used to establish the binding mode-predicting workflow were extracted from the IGF1R crystal structure complexes collected from the PDB database. Bond orders were assigned, H-atom and gasteiger-atom partial charges were added for extracted ligands, and data were saved in a mol2 file. Main physicochemical properties were calculated using ChemMine Tools [49], and molecular weight and fraction of rotatable bonds were chosen as the criteria for clustering after theorizing and testing. Crystal structures of IR complexes were also collected from the PDB database, choosing only those that contained nonprotein compound ligands without phosphate groups. Ligands were extracted from these complexes, and prepared and clustered as for IGF1R.

#### 3.3.2. Binding Mode Predictions

##### 3.3.2.1. Ligands with small *MW* (*MW* ≤ 440) and low FLEX (FLEX ≤ 0.16)

These ligands were processed by surflex to generate possible binding conformations. Additional starting conformations for each molecule and search density were set to 6. The search grid was expanded to 6 ångstroms, and the conformations per fragment were set to 20 or lower. In addition, the number of rotatable bonds per molecule was set to 100 or fewer. The activated spin alignment method was used to orient the ligand, with the search density set to 6 and number of spins set to 12. For each ligand, the top-ranked five conformations were saved with a minimum RMSD of 1.0 between any two consecutive conformations. The saved data were re-ranked by DOCK AMBER [50]. In DOCK AMBER, only ligands were allowed to move while receptors were frozen. For other parameters, default settings were adopted [51]. Finally, the top-ranked conformation was selected as the most probable.

##### 3.3.2.2. Ligands with small *MW* (*MW* ≤ 470) and high FLEX (FLEX > 0.16)

The re-docking workflow for this type of ligand was mainly the same as that of the former type, with some differences: by Surflex calculation, the search density and number of spins were increased to 9 and H-atoms of receptor active sites were allowed to move. For each ligand, the top-ranked 10 conformations were saved with a minimum RMSD of 1.0 between any two consecutive conformations. In DOCK AMBER, ligands and residues within 4.0 ångstroms of user-customized “match spheres” were allowed to move. The number of conjugate gradient minimization cycles performed before or after MD were increased to 150, and the number of MD steps was increased to 3500. The definition of “match spheres” was carefully checked to ensure that residues with low flexibility were not identified as movable in the MD procedure. The top-ranked conformation was selected as the most probable.

##### 3.3.2.3. Ligands with small *MW* (440 < *MW* ≤ 470) and low FLEX (FLEX < 0.16)

This type of ligand was processed by Vina to generate possible binding conformations. Superimposition of available receptor crystal structures and the b-factor distribution of receptor atoms were inspected carefully to identify residues with side chains that were allowed to move. The grid box was adjusted to reduce the size to 22.5 ångstroms in each direction, and the exhaustiveness was increased to 24. Energy differences between the best and worst mode were set to 4. The top-ranked five binding modes were re-ranked by DOCK AMBER. In the MD procedure, ligands and residues within 5.0 ångstroms of user-customized “match spheres” were allowed to move. The top-ranked conformation was selected as the most probable.

##### 3.3.2.4. Ligands with large *MW* (*MW* > 470)

For this type of ligand, Vina was employed to generate a set of 20 possible conformations, and DOCK AMBER was used to select the best one. Most settings were as described for ligands with large M.W. and low Flex, except that in Vina, the exhaustiveness was increased to 48, and the top 20 modes were processed by DOCK AMBER. MD steps were increased to 3500 and energy minimizations were increased to 150.

For each pair of re-docked and crystal structure ligands, the RMSD between their original positions was calculated to evaluate the degree of superimposition. ProFit [52] was employed to complete calculations.

### 3.4. Virtual Screening of NCI Database

#### 3.4.1. Preparations of Protein Receptors and Dockable Ligand Database

The PDB database contains many crystal structures of IGF1R with different conformations near the active site, as this is a high flexibility region in kinases. We chose PDB ID 2OJ9 because of its high resolution. 2OJ9 also provides an appropriate size of the active site to accommodate most known inhibitors. Preparation of the 2OJ9 protein was as in section 3.1. Similarly, the IR complex structure PDB ID 3BU3 was used for comparison of specific IGF1R inhibitors. Preparation of IR was as for IGF1R.

The ligand database was established based on a pool of 139,735 compounds in SD format from DTP [17]. Compounds were filtered by FILTER of OpenEye, using “blockbuster” solution with several parameters altered to relax restrictions. Qualified compounds were sent to QUACPAC to calculate possible proton states from pH 5.8 to 8.2 and tautomers. Gasteiger-Marsili partial charges were added. A mol2 file containing the produced structures was processed by OMEGA for sample conformations for each ligand.

#### 3.4.2. Identification of Potential Specific Inhibitors of IGF1R

The virtual screening workflow was used to screen the NCI database to identify potential IGF1R inhibitors. Selected compounds were used in the binding mode-predicting workflow to predict modes of binding in the ATP-binding site of IGF1R and IR. For each ligand, the binding modes for IGF1R and IR were compared. Potential specific IGF1R inhibitors were identified according to the rules in Section 2.3.

### 3.5. PMF Calculations with GROMACS

MD simulations used GROMACS software [40]. VMD software was used for system constructions and results analysis [53]. Proteins were prepared in the amber03 force-field, and ligands were prepared using Amber Tools [54]. The struture of each receptor-ligand complex was solved in the TIP3P water model, and Na^+^ or Cl^−^ ions were added to ensure an overall system charge of 0. Periodic boundary conditions were added to all MD simulations. Solved complexes were subjected to a 50,000-step energy minimization using a steep decent algorithm, followed by a 500 ps equilibration in an NPT (N: number of moles, P: pressure, T: temperature) ensemble. The time step was set to 2 fs for all simulations. Pressure was maintained at 1.0 bar using the Berendsen method and temperature was kept at 310 K using the Berendsen integrating method. Electronic interactions were computed with the particle-mesh Ewald (PME) algorithm. The cutoff for nonbonded interactions and the distance for Lennard-Jones interactions was 14 ångstroms. Trajectory data were written for each 1 ps. Positions of complex atoms were restrained by a force constant of 1000 kJ/nm^2^·mol in *x*, *y* and *z* directions. After NPT equilibration, the ligand was steered out of its receptor binding site using harmonica force with a spring constant of 800 kJ/nm^2^·mol, and the pulling rate was set to 0.8 nm/ns. Along the reaction axis, umbrella sampling windows were set at intervals of roughly 0.1 nm. For each umbrella sampling window, an NPT equilibration was performed followed by umbrella sampling production, with either procedure lasting for 500 ps. The PMF was calculated from the umbrella sampling [55] results using the weighted histogram analysis method (WHAM) [56,57].

## 4. Discussion

Flexible docking, especially involving conformational flexibility of a protein backbone, is a challenge because of the large conformational space to be sampled [16]. Existing software has only partially solved this problem. In this study, because of the high flexibility of the active site of IGF1R, we established a virtual screening workflow for identifying IGF1R inhibitors through the best use of optional functions for flexible docking in existing software. For example, in Surflex, turning on the “protein flexibility” switch allowed receptor H-atoms to move. The latest versions of Autodock and Vina allow side chains of chosen residues to move to simulate a flexible binding site. In DOCK, allowing more atom clashes between ligand and receptor might partially solve problems of flexible docking. However, problems still exist. For Autodock, only 32 or fewer torsions are allowed, so having movable side chains on about 10 residues could lead to errors. Although Vina has no restrictions on torsion number, stable results were rare when the exhaustiveness was increased from a default of 9 to 24 or 48. Our virtual screening workflow was created mainly based on benchmark results of several popular open/free docking packages. More extensive benchmark testing on other open/free and commercial software is still needed.

Ligands with many rotatable bonds could be a challenge to docking software because of the increased difficulty in predicting correct ligand binding positions [58]. This motivated us to create the binding mode-predicting workflow that accounted for the physiochemical characteristics of ligands. MD is a good method for studying molecular interactions, including the analysis of ligand-receptor binding conformations. MD has been implanted into docking packages such as the AMBER score in UCSF DOCK. However, because of the heavy calculation burden of AMBER scoring during MD simulation, using the AMBER score for virtual screening of a large ligand database is impractical. In our workflow, we adopted different docking strategies to predict binding modes of ligands, according to ligand clustering. In particular, the AMBER score was introduced for ligands with high molecular weight and high flexibility. Because of the limited number of available ligands, our clustering analysis was mainly dependent on molecular weight and fraction of rotatable bonds. This simple method might not be appropriate for all ligands and receptors. Further improvements are needed, such as finding additional characteristics for grouping ligands correctly.

Another challenge for IGF1R is the discovery of lead compounds that can inhibit IGF1R but not IR. Reported compounds with high selectivity for inhibition of IGF1R over IR were found by experimental approaches, and the mechanism of their selective inhibition was thought to be conformational differences. Based on our comprehensive analysis of those specific IGF1R inhibitors, we proposed several stringent rules for identifying specific IGF1R inhibitors from the potential compound set obtained by the two workflows. Moreover, we performed steered molecular dynamics simulations to calculate the PMF with GROMACS for the most potent compounds. The simulation results confirmed the effectiveness of our approaches. The binding affinities of the identified ligands to IGF1R will be determined by experimental verification.

## 5. Conclusions

This study established a receptor-based virtual screening workflow and a binding mode-predicting workflow for the identification of specific ATP-competitive inhibitors of IGF1R that are not specific to IR. Both workflows used free academic software so they are widely transferable and can be developed by other users. Although the two workflows were built for virtual screening of IGF1R inhibitors, virtual screening of other receptors with highly flexible active sites should be possible by adjusting software parameters and workflow components according to benchmark results. Using the two workflows, 17 potential lead compounds were identified as specific inhibitors of IGF1R from about 140,000 candidates in the NCI database. Among the 17 compounds, 351570, 660826 and 649812 were considered powerful specific IGF1R inhibitors. Screening results were tested by PMF calculations, and 351570 and 649812 were confirmed to show higher binding affinity on IGF1R than on IR.

## Supplementary Information



## Figures and Tables

**Table 1:** Average screening time taken by chosen software (computing environment: Dell precision T7500 workstation with 12 core E5620 cpu and 8G RAM). The second column indicates the time taken for per molecule in average (unit: second), and the third column predicted total time for calculation of 100,000 molecules, as well as the fourth column transformed time unit from seconds to days for the third column.

Software/time	Per (s)	Total (s)	Days (d)
Fred	0.415	41500	0.480
Surflex	7.461	746100	8.635417
Dock	8.474	847400	9.80787
Autodock	94.787	9478700	109.7072
Vina	33.497	3349700	38.76968

**Table 2:** Values of molecular weight and fractions of rotatable bonds of the nine crystal ligands and characteristics of four ligand types after clustering. In-place root-mean-square deviation (RMSD) values of re-docked ligands were listed in the last column. “Mismatch” indicates that there is no suitable predicting poses available.

PDB.ID	Molecular weight	Fraction of rotatable bonds	RMSD value (ångstroms)
2OJ9	380	0.147	1.104
2ZM3	406	0.133	1.250
3LVP	329	0.111	0.218
3QQU	404	0.167	1.866
3I81	438	0.179	3.836
4DCE	459	0.256	4.071
3D94	460	0.093	1.512
3O23	448	0.111	Mismatch
3F5P	524	0.293	Mismatch

**Table 3:** Values of molecular weight and fractions of rotatable bonds of the four insulin receptor (IR)-crystal ligands and characteristics of ligand types after clustering. In-place RMSD values of re-docked ligands were listed in the last column. “Mismatch” indicates that there is no suitable predicting poses available.

PDB.ID	Molecular weight	Fraction of rotatable bonds	RMSD value (ångstroms)
3EKN	443	0.190	1.207
3ETA	499	0.200	3.693
3EKK	504	0.233	4.065
2Z8C	358	0.233	Mismatch

**Table 4:** The binding energies of the three compounds to IGF1R and IR and the known ligand to IGF1R (2OJ9).

Receptor name	Compound ID	ΔG_bind_ (kCal mol^−1^)
IGF1R	351570	−13.5
	660826	−3.09
	649812	−5.92
	2OJ9	−4.38
IR	351570	−7.2
	660826	−5.39
	649812	−0.60
