# Supplementary material for: Virtual Screening of Specific Insulin-Like Growth Factor 1 Receptor (IGF1R) Inhibitors from the National Cancer Institute (NCI) Molecular Database
Source: Int J Mol Sci. 2012 Dec 14;13(12):17185–209. doi: 10.3390/ijms131217185 (PMC3546745; doi:10.3390/ijms131217185)
Supplement: Supplementary file 1 [file ijms-13-17185-s001.pdf]

Supplementary Information

Figure S1. The detail results on the distribution of enriched active ligands calculated for each benchmarked software. Horizontal axis indicates top percentage of rank, Vertical axis indicates percentage of selected ones in total known binding ligands.

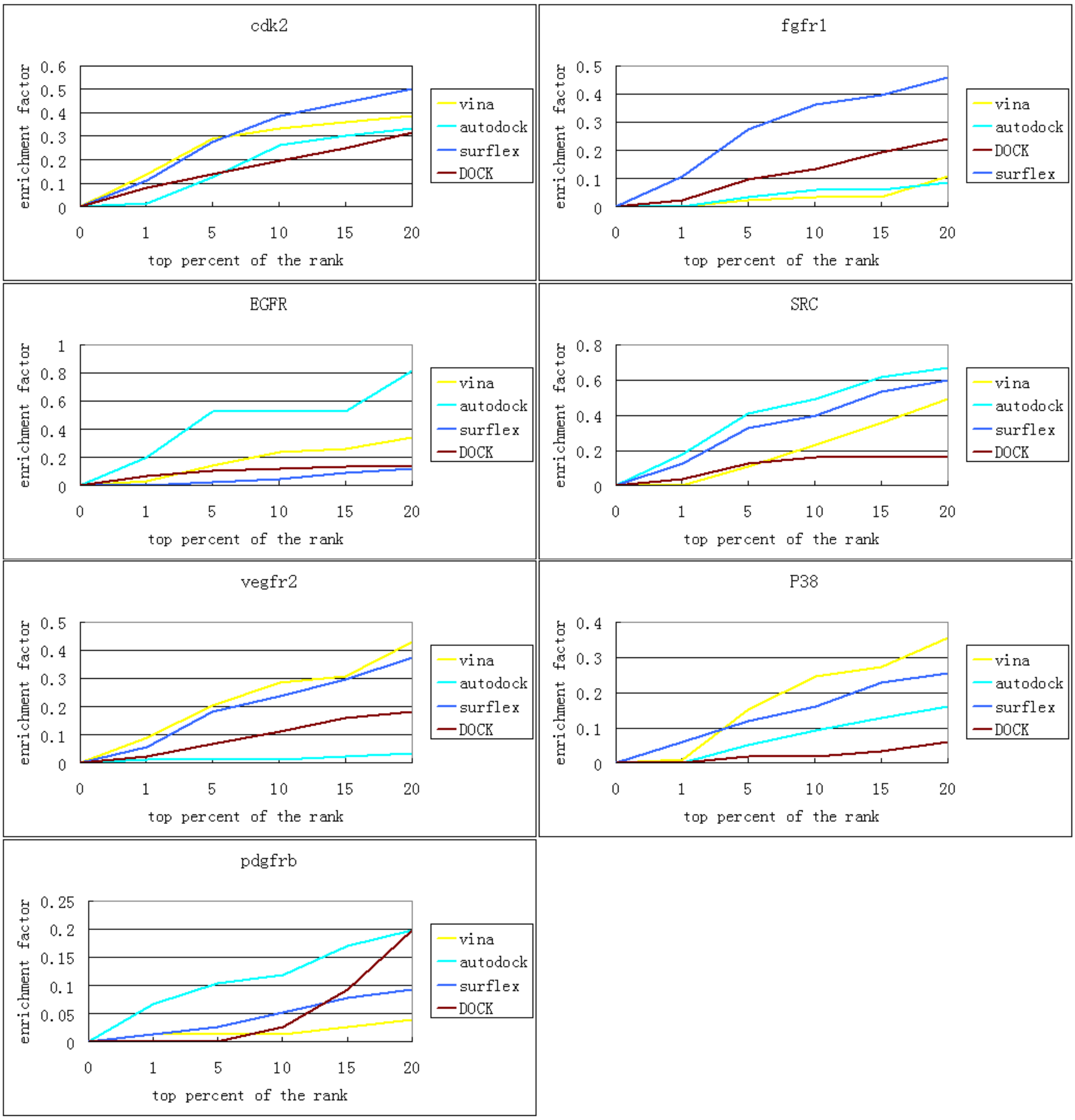

Figure S2. Comparisons of binding pose respectively against IGF1R and IR for each identified IGF1R specific inhibitors. Atoms were colored by the type. Protein residues were represented by lines and ligands were represented by sticks. Green lines and sticks exhibit ligands' binding pose in IGF1R active site, while cyan ones exhibit ligands' binding pose in IR active site. Yellow dash lines indicate H-bonds automatically exhibited by Pymol while the one with a number on it indicate H-bond that was not identified by Pymol [25]. For compound named 640627, no reasonable pose was generated in the active site of IR.

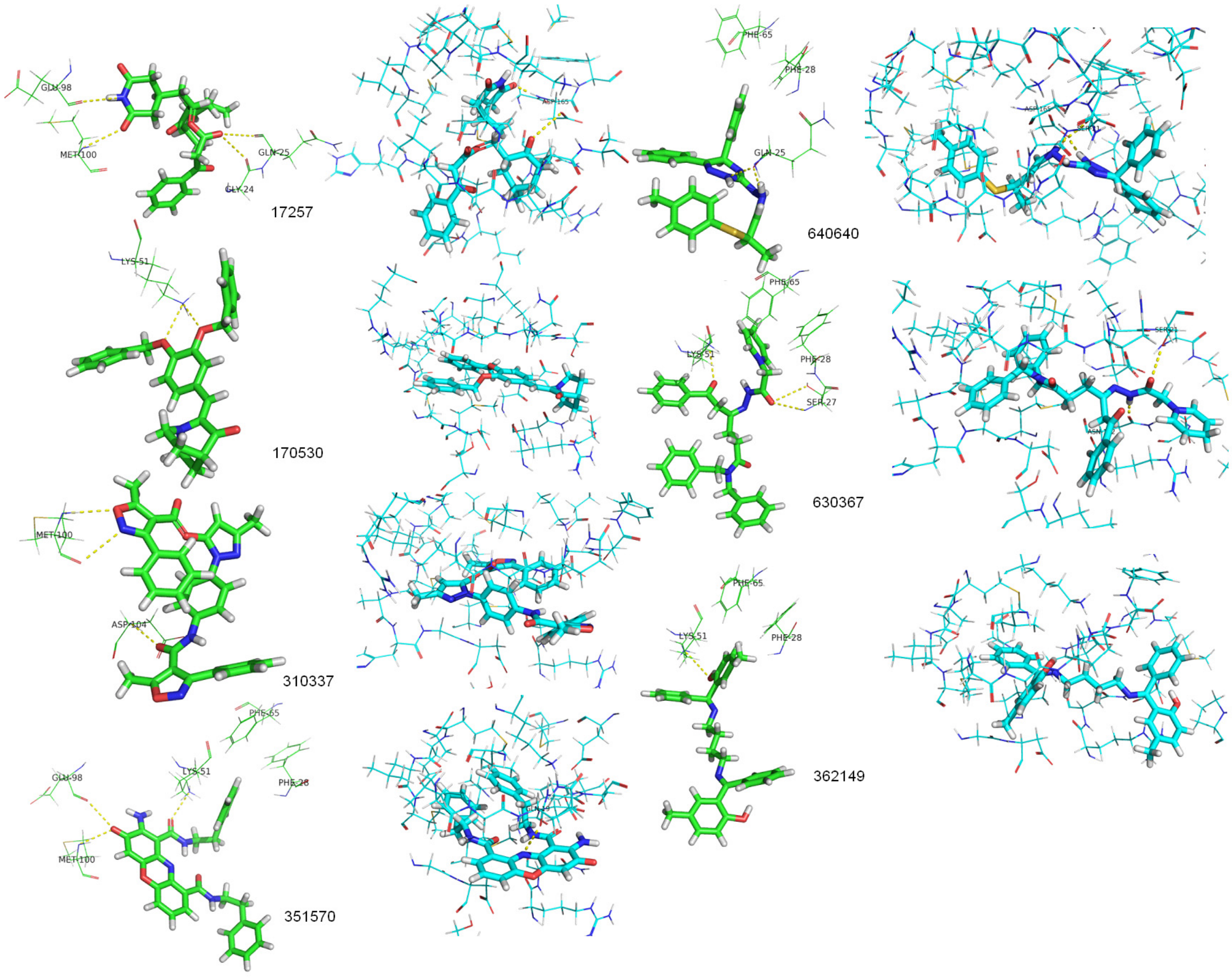

Figure S2. *Cont.*

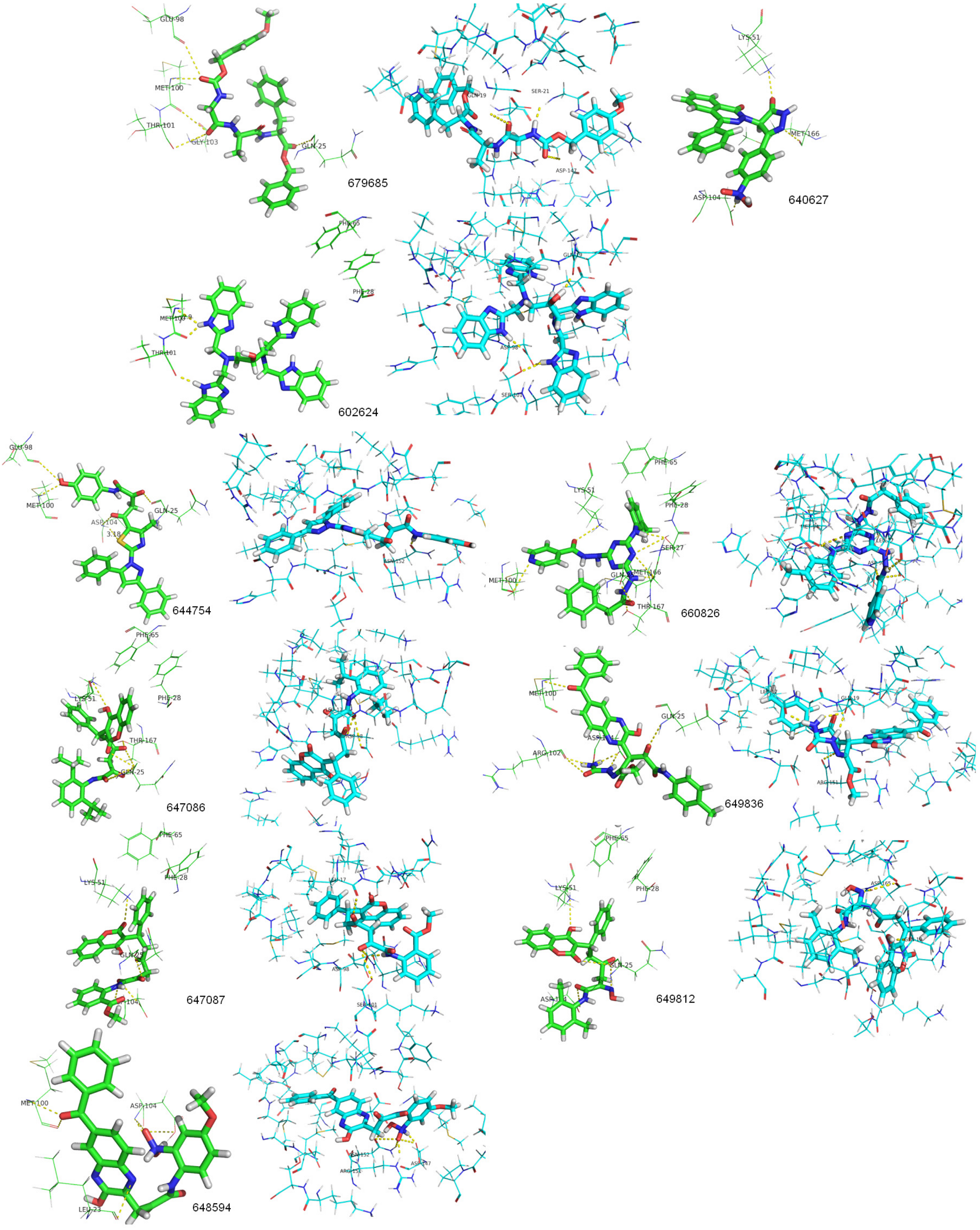

© 2012 by the authors; licensee MDPI, Basel, Switzerland. This article is an open access article distributed under the terms and conditions of the Creative Commons Attribution license (http://creativecommons.org/licenses/by/3.0/).
